# *Rosmarinus officinalis L.* (Rosemary) Extracts Containing Carnosic Acid and Carnosol are Potent Quorum Sensing Inhibitors of *Staphylococcus aureus* Virulence

**DOI:** 10.3390/antibiotics9040149

**Published:** 2020-03-31

**Authors:** Seitaro Nakagawa, Greg G. Hillebrand, Gabriel Nunez

**Affiliations:** 1 Department of Pathology and Rogel Cancer Center, University of Michigan Medical School, Ann Arbor, MI 48109, USA; snakagaw@med.umich.edu (S.N.); bclx@med.umich.edu (G.N.); 2 Department of Innovation and Science, Amway Corporation, Ada, MI 49355, USA

**Keywords:** skin microbiome, *Staphylococcus aureus*, *agr*, quorum sensing inhibition, atopic dermatitis, anti-virulence therapy

## Abstract

*Staphylococcus aureus* is an opportunistic pathogen and a common cause of skin infection. *S. aureus* also plays a role in the pathogenesis of the chronic inflammatory skin disease, atopic dermatitis. *S. aureus* virulence involves activation of the quorum sensing *agr* operon. In this paper, we show that the diterpene carnosic acid, present in *R. officinalis L*. (rosemary) leaves, is a specific inhibitor of *S. aureus agr* expression as low as 5 μM. Carnosol and rosmarinic acid are two other phytochemicals present in rosemary leaves. Carnosol, but not rosmarinic acid, is also a potent *agr* expression inhibitor. Natural rosemary extracts containing carnosic acid and carnosol inhibit *S. aureus agr* expression, both in luciferase reporter strains and in wild type strains isolated from patients with atopic dermatitis. Specific inhibition of *S. aureus* virulence using topical formulations of rosemary extract may offer a practical approach to preventing and treating flares of atopic dermatitis.

## 1. Introduction

*Staphylococcus aureus*, a Gram-positive bacterium, can reside as a normal friendly commensal in up to two-thirds of healthy individuals [1]. However, *S. aureus* may be better known for its sinister side as one of the ESKAPEE opportunistic pathogens causing serious life-threatening infections, most notably those infections with methicillin-resistant *S. aureus* (MRSA) [2,3]. This “Jekyll and Hyde” personality of *S. aureus* may play a role in non-infectious disease as well. Atopic dermatitis (AD) is a chronic inflammatory skin disease characterized by recurring flares of erythema, edema, scaling, and itch. It affects 15–30% of children and approximately 5% of adults in industrialized countries [4,5]. More than 90% of AD patients are colonized on the lesional skin by *S. aureus* [6] and *S. aureus* colonization and biofilm formation are directly associated with disease flares and remission [7,8]. Although treatment with lukewarm bleach baths and steroid creams or immunosuppressive, anti-histamine or anti-IL-4R drugs are approved for AD patients [9,10,11,12], most of these treatments have limited effectiveness and negative side effects. An alternative treatment approach is to specifically target the mechanism of AD pathogenesis which should lead to more effective prophylactic and therapeutic solutions without the negative side effects. 

Quorum sensing is the mechanism by which bacteria sense, communicate and respond to their own cell density. This cell-to-cell communication system is used by bacteria to regulate a variety of physiological functions via activation of genes involved in virulence and biofilm formation [13,14]. *S. aureus* uses quorum sensing to invade tissues of the human body including skin [8]. The operon controlling quorum sensing in *S. aureus* is called the accessory gene regulator or *agr* [15,16]. *Agr* might be thought of as the “Jekyll and Hyde gene”, regulating the dual personalities of *S. aureus*. Activation of *agr* causes the transformation of the friendly and commensal “Dr. Jekyll” *S. aureus* into the evil and virulent “Mr. Hyde” *S. aureus*.

The *agr* system of *S. aureus* contains two transcriptional units, RNAII and RNAIII that are transcribed in opposite orientation [17]. In the *agr* pathway, the cyclic thiolactone peptide pheromone called autoinducing peptide (AIP) generated from the AgrD precursor is released by the export protein AgrB and activates the AgrC/AgrA two-component signal transduction system. Upon binding of the AIP to the receptor kinase AgrC, the response regulator AgrA is activated and binds to the promoter regions in the *agr* system (P2 for RNAII and P3 for RNAIII) [18]. The production of RNAII and RNAIII leads to the production of virulence factors including the phenol soluble modulins (PSMs), a group of cytotoxic peptides that are important for the outcome of infections by community-associated MRSA and skin inflammation in a feedback loop [19]. 

δ-toxin is a 26-amino acid PSM that stimulates rapid mast cell degranulation and release of histamine [20]. Another PSM produced by *S. aureus* during quorum sensing, PSMα, can promote skin inflammation in vitro in human keratinocytes [21]. Using a mouse model of epicutaneous *S. aureus* infection, Nakamura et al. found that δ-toxin and PSMα promote AD-like skin inflammation [20]. Finally, expression of RNAIII is increased in the skin lesions of children with AD [20]. The potential role of *agr* expression and the quorum sensing system in AD etiology makes it an attractive mechanistic target for treatment intervention. 

Quorum sensing inhibition or QSI has been proposed as an alternative therapeutic approach to conventional antibiotics. Since the target of QSI is bacterial virulence, not viability, there is less chance that the bacteria will develop resistance to the quorum sensing inhibitor [22,23]. The therapeutic potential of targeted anti-virulence therapy was demonstrated using Solonamide B. Solonamide B is a cyclodepsipeptides isolated from a marine bacterium *Photobacterium spp*. with a tertiary structure remarkably like AIP. Due to this structural similarity, Solonamide B is a competitive inhibitor of AIP and interferes with AIP binding to AgrC, the sensor kinase. Solonamide B blocks δ-toxin synthesis and reduces *S. aureus*-induced skin inflammation in an epicutaneous mouse model [24]. More recently, Williams et al. showed that certain commensal strains of *S. hominis* produce an AIP that interferes with *S. aureus agr* toxin production and inflammation in murine skin [25].

**Figure 1:**
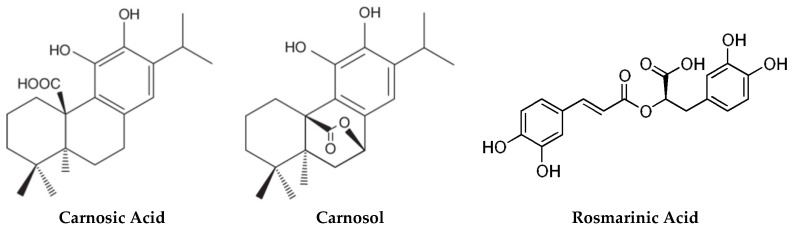
Major phytochemicals in rosemary.

The mechanisms of bacterial virulence can be shared across plants and animals [26,27]. As such, plants have evolved sophisticated host defense mechanisms to prevent bacterial infection, many of which may have utility in human medicine. These include the terpenes and flavonoids present in plant essential oils [28,29]. Carnosic acid is a diterpene present in high levels in *Salvia* and *Rosmarinus* plant species [30]. Certain *Rosmarinus officinalis* L. (rosemary) cultivars can carry as much as 10% carnosic acid in the air-dried leaves. In the plant, carnosic acid helps to protect chloroplasts and chloroplastic membranes from oxidative stress [31,32]. Extracts of rosemary have a long history of medicinal use [33]. For example, topically applied rosemary extract accelerates wound healing in a mouse model [34] and is used in the treatment of androgenic alopecia [35]. Rosemary extract contains in addition to carnosic acid, carnosol and rosmarinic acid both of which have antimicrobial, antioxidant and anti-inflammatory properties [33,36] (Figure 1).

Nunez et al. used a high throughput in vitro assay to screen over 4000 compounds for their ability to inhibit *S. aureus agr* expression [37]. Carnosic acid at 10 μM was identified as one of the most potent compounds. Here we extend that observation to characterize carnosic acid and rosemary extracts for inhibiting *agr* expression.

## 2. Results

### 2.1. Carnosic Acid and Carnosol, but not Rosmarinic Acid, Specifically Inhibit agr Virulence Expression

**Figure 2:**
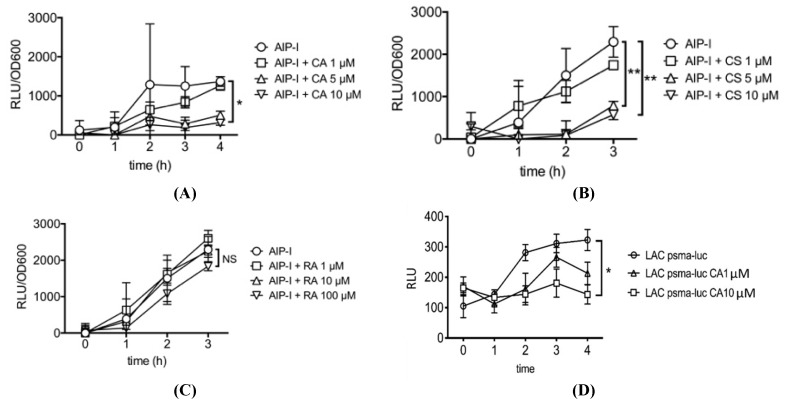
Effect of (**A**) carnosic acid (CA), (**B**) carnosol (CS) (**C**) rosmarinic acid (RA) on AIP-induced *S. aureus* agr RNAIII gene expression relative to total bacterial density (OD600). (**D**) Effect of carnosic acid (CA) on *psm*α gene expression. Data are presented as mean ± SD. *p < 0.05, **p < 0.01.

Figure 2 shows the time course and dose response for inhibition of AIP-induced *S. aureus agr* RNAIII gene expression, relative to total bacterial density (OD600), by carnosic acid, carnosol and rosmarinic acid. Carnosic acid showed significant and specific inhibition of AIP-induced RNAIII expression as low as 10 μM and carnosol showed significant inhibition as low as 5 μM (Figure 2A,B). Rosmarinic acid (RA) on the other hand did not significantly inhibit AIP-induced RNAIII expression when tested up to 100 µM (Figure 2C). We also tested carnosic acid, carnosol and rosmarinic acid for inhibition of AIP-induced *psm*α gene expression using a *psm*α*-lux* reporter strain. Similar to the results for RNAIII, carnosic acid and carnosol, but not rosmarinic acid, significantly inhibits *psm*α gene expression at concentrations as low as 10 μM. Figure 2D shows the results for carnosic acid inhibition of AIP-induced *psm*α gene expression. In other experiments not shown, we compared the combination of carnosic acid plus carnosol vs. each compound alone for inhibition of RNAIII expression. No apparent synergy for the combination was observed. Similarly, no synergy was observed for the combination of carnosol plus rosmarinic acid.

### 2.2. Rosemary Extracts Containing Carnosic Acid Specifially Inhibit RNAIII agr Virulence Expression

**Figure 3:**
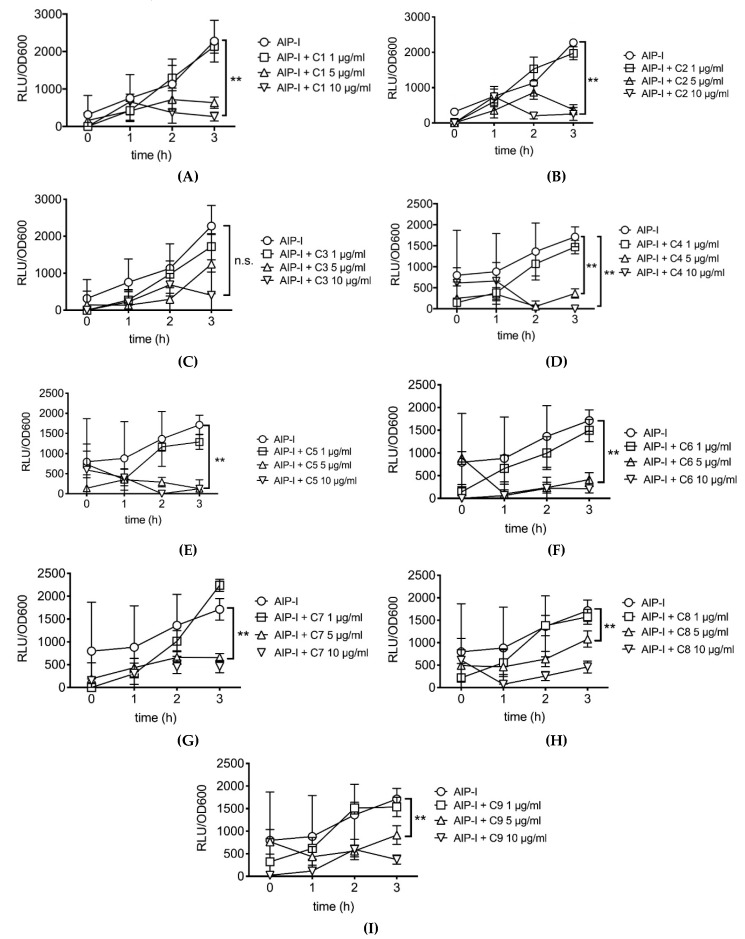
Dose response of AIP-induced RNAIII inhibition by rosemary extracts C1–C9 ((**A**–**I**), respectively). See Table 1 for carnosic acid, carnosol and rosmarinic acid concentrations in each extract. Data are presented as mean ± SD. *p < 0.05, **p < 0.01.

Because carnosic acid and carnosol are phytochemicals present in rosemary, we tested 9 different rosemary extracts containing various concentrations of carnosic acid and carnosol for inhibition of *agr* virulence gene expression in *S. aureus*. Table 1 shows the concentrations of carnosic acid, carnosol and rosmarinic acid in each of the tested extracts. All the rosemary extracts except C3 significantly inhibited AIP-induced RNAIII gene expression as low as 5 µg/mL (Figure 3). Extract C3 inhibited RNAIII expression but the variability in this particular experiment did not allow the differences to achieve statistical significance.

### 2.3. Carnosic Acid, Carnosol, and Rosemary Extracts Inhibit RNAIII and psmα Gene Expression in Clinical Strains of *S. aureus* from Atopic Dermatitis Patients

**Figure 4:**
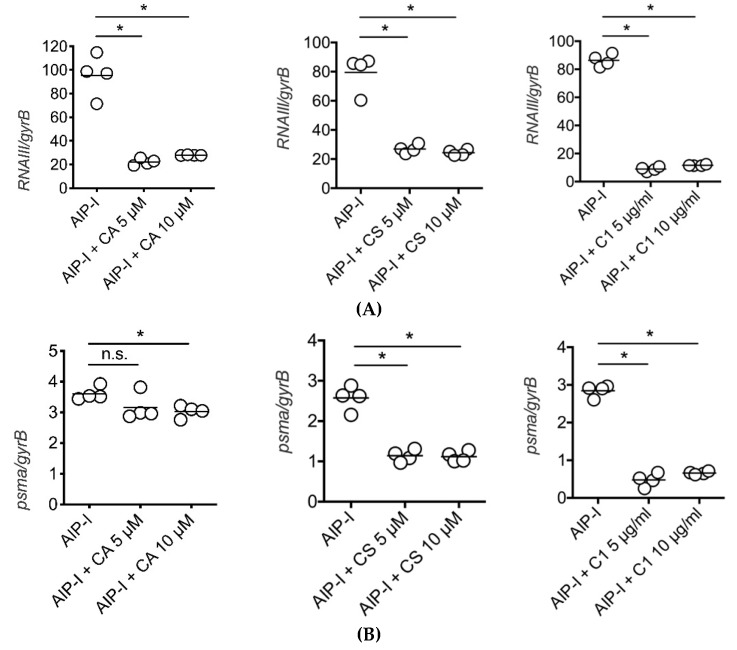
qPCR determination of (**A**) RNAIII and (**B**) psmα gene expression inhibition by (left to right) carnosic acid (CA), carnosol (CS) and rosemary extract (C1) in an AD clinical strain of *S. aureus*. Bars represents mean of each group. *p < 0.05, by Mann–Whitney two-tailed *t*-test. n.s., not significant.

We used quantitative real-time PCR (qPCR) to determine if pure carnosic acid, carnosol and rosemary extracts could inhibit AIP-induced RNAIII and psmα gene expression in clinical strains of *S. aureus* isolated from patients with atopic dermatitis. We selected one of the most virulent *S. aureus* strains as determined by cytotoxicity for these experiments. Three hours after we stimulated the clinical strain with type I AIP (50 nM) in the presence of 5 or 10 μM carnosic acid or carnosol or 5 or 10 μg/mL rosemary extract, we collected the bacteria and immediately isolated mRNA and then measured RNAIII and psmα expressions relative to the *S. aureus* housekeeping gene, gyrB. As we observed in the luciferase gene reporter strain of *S. aureus*, carnosic acid and carnosol as well as the rosemary extract specifically inhibited RNAIII and psmα virulence gene expression in the clinical strain isolated from AD patients (Figure 4). 

## 3. Discussion

Bacterial pathogens have numerous virulence mechanisms to allow them to enter, replicate within, and persist at the host sites [38] but with only a few common mechanistic themes [39]. The virulence factors produced by pathogenic organisms such as proteases, toxins, and biofilms, are largely responsible for the damage caused to the host tissue. As such, inhibiting these virulence factors, also known as anti-virulence technology, has emerged as an attractive therapeutic alternative to antibiotics [17,19,40]. It is supposed that by specifically targeting the mechanisms of bacterial virulence, and not viability, the pathogenicity of the bacteria can be controlled without increasing the chance of developing a resistant phenotype. Moreover, by specifically targeting the virulence mechanisms, there is less chance of disrupting the composition of the commensal or beneficial microorganisms in and on the host. 

Bacterial quorum sensing is a key mechanism of bacterial virulence. Quorum sensing is the cell-cell communication system used by bacteria to sense their population density and adapt to the environment [13,15]. There are several approaches to the design of effective therapeutics based on quorum sensing inhibition. Many directly and indirectly target inhibition of the amount or function of the peptide auto-inducers. Such inhibitors can be found in nature with Solonamide B as a perfect example [41]. Since many host defense responses to bacterial pathogens are common to plants, insects and animals, it becomes obvious to look at plant-based small molecules as a rich source for new therapeutic agents based on quorum sensing inhibition [27,29]. 

The use of flavonoids and other small molecules from plants as anti-virulence agents has been reported previously and reader is directed to the excellent review by Wu et al. [42]. Dong et al. showed that the flavonoid morin, used in traditional Chinese medicine, directly inhibits the hemolytic activity of aerolysin, the primary virulence factor of *A. hydrophila* strains and protects catfish from *A. hydrophila* infection [43]. Eugenol, a constituent of clove oil, can inhibit quorum sensing in methicillin-resistant *S. aureus* (MRSA) clinical strains [44]. Low concentrations of tea tree oil (from M. alternifolia) containing terpinen-4-ol inhibit MRSA biofilm formation [45]. Essential oil from *S. hortensis*, containing carvacrol, terpinene and thymol, significantly down-regulates the *S. aureus hld* gene expression at concentrations below the bacterial minimal inhibitory concentration [46]. The triterpene saponin glycyrrhetinic acid isolated from liquorice is a potent inhibitor of *S. aureus* alpha-haemolysin (Hla) at 1-8 μg/mL, concentrations well below its minimal inhibitory concentration (MIC) of >512 μg/mL and protects mice from *S. aureus* pneumonia [47]. Hydroalcoholic extracts of rosemary, similar to the extracts used in our study, have been shown to exhibit anti-MRSA biofilm activity as low as 20 μg/mL [48]. De Olivira et al. have also found that rosemary extracts to be a safe and effective in controlling not only *S. aureus*, but also *Candida albicans*, *Enterococcus faecalis*, *Streptococcus mutans*, and *Pseudomonas aeruginosa* in both mono- and polymicrobial biofilms. They suggested that extracts of rosemary should have potential as a therapeutic agent when added to formulations such as toothpastes, skin creams and soaps [49]. 

In the present study, we have extended our understanding for how rosemary extracts may impart anti-virulence biological activity. We have shown that two primary constituents of rosemary leaves, in pure form, carnosic acid and carnosol, are potent and specific inhibitors of *S. aureus* RNAIII and *psmα* gene expression. Rosmarinic acid, a water-soluble rosemary phytochemical, was shown to be inactive in these assays. Nine different hydroalcoholic extracts of rosemary, with analytically determined amounts of carnosic acid and carnosol, also inhibited *S. aureus* virulence expression. Importantly, we observed this anti-virulence activity both in luciferase reporter strains of *S. aureus* as well as in clinical isolates from atopic dermatitis patients using direct qPCR quantification of gene expression.

The concentration of pure carnosic acid or carnosol required to specifically inhibit *S. aureus* RNAIII and *psmα* gene expression is in the low μM range (≥5 μM). The rosemary extracts tested were also potent RNAIII and *psmα* gene expression inhibitors showing significant inhibition in the low μg/mL range (≥5 μg/mL). At 5 μg/mL, the total molar concentration of carnosic acid plus carnosol in Extract C2, is 2 μM, consistent with the results observed with the pure compounds. At these concentrations, there is little to no inhibition of bacterial growth. Endo et al. measured the minimal inhibitory concentration (MIC) of a hydroalcoholic extract of *R. officinalis* for inhibition of various strains of MRSA in both planktonic and biofilm states [48]. Rosemary extracts showed MICs ranges from 15.6–62.5 μg/mL for planktonic growth and as low 45-250 μg/mL for preformed biofilms, depending on the MRSA isolate.

In theory, the *S. aureus* bacteria present in and on AD skin lesions should come into direct contact with the topically applied formulation. That is, little if any penetration of carnosic acid through the stratum corneum barrier (compromised or not) should be necessary. This would imply that the concentration of carnosic acid in a topical formulation required to specifically inhibit *agr*, without killing the bacteria, should be similar to those observed in the in vitro experiments presented in this paper. This is an important consideration as we try to translate our lab results to clinical application. 

## 4. Conclusions

Plants and animals have evolved similar host defense strategies to fight bacterial pathogens. As a result, plant extracts contain potent antimicrobial and anti-virulence compounds with tremendous potential as novel safe and effective drugs as well as utility in cosmetic products. In this study, we characterized pure carnosic acid, carnosol and rosmarinic acid, compounds found in *R. officinalis*, for inhibiting the *agr* quorum sensing pathway involved in *S. aureus* virulence associated with the atopic dermatitis. As a next step, we plan to clinically test topical formulations of a natural rosemary extract for preventing and treating flares of atopic dermatitis.

## 5. Materials and Methods 

### 5.1. Strains Utilized 

For RNAIII gene expression, we used the LAC P3-lux strain and for *psmα* gene expression we used the psmα-lux strain as reported previously [20,50]. *Psmα* luciferase gene reporter LAC *Staphylococcus aureus* (pulsed-field type USA300) strains were kindly gifted from Dr. Michael Otto at the National Institutes of Health. Clinical isolates of *Staphylococcus aureus* strains from children diagnosed with atopic dermatitis were collected originally from the Department of Laboratory Medicine and Pathology at University of Toronto.

### 5.2. Test Materials

Pure carnosic acid, carnosol and rosmarinic acid standards were purchased from Sigma-Aldrich (St. Louis, MO, USA). Rosemary (*R. officinalis* L; *Lamiaceae*) extracts were from 1) Naturex (C1, Avignon Cedex, France), 2) Euromed (C2, Mollet des Valles, Spain) and 3) Amway Corporation (C3-C9, Buena Park, CA). The weight:weight percentage concentrations of carnosic acid, carnosol and rosmarinic acid in each of the 9 rosemary extracts tested are shown in Table 1. Concentrations for the extracts from Naturex and Euromed reflect the vendor’s certification of analysis. Concentrations for all other extracts were determined by ultraperformance liquid chromatography (UPLC). All test materials were dissolved in DMSO immediately before each experiment.

### 5.3. Ultraperformance Liquid Chromatography

Primary analytical reference standards were purchased from ChromaDex Corp. (Los Angeles, CA). Rosemary extract samples were accurately weighed and dissolved in isopropyl alcohol, vortexed and sonicated for 15 minutes to allow for full extraction. Samples were then filtered (0.22 μm) and submitted for UPLC using a Waters Acquity H-Class UPLC instrument equipped with an Acquity e-lambda photodiode array detector. The detection wavelength was 280 nm and Empower 3 software (Waters) was used to analyze the data and calculate assay results. The column used was a Waters Acquity UPLC HSS T3, 1.8 μm, 2.1 × 100 mm and the mobile phase employed a ternary gradient of (A) 0.1% trifluoroacetic acid in water, (B) 0.1% trifluoroacetic acid in methanol, and (C) 0.1% trifluoroacetic acid in acetonitrile. 

### 5.4. Reporter Assay

Gene reporter strains were cultured in tryptic soy broth (TSB) and grown to mid-exponential growth assessed by optical density at 600 nm (OD600). The cultures (n=5 per treatment) were then stimulated with recombinant type I AIP (50 nM) with or without the various test materials. Light emitted by the luciferase gene reporter fusion constructs was measured using a Synergy HT (BioTek, Winooski, VT, USA) and reported at relative light units (RLU). Data are expressed as a ratio of RLU to OD600. Three hours after we stimulated the clinical strain with type I AIP (50 nM) in the presence of 5 or 10 μM carnosic acid or carnosol or 5 or 10 μg/mL rosemary extract, we collected the bacteria and immediately isolated mRNA and then measured RNAIII and psmα expressions relative to the *S. aureus* housekeeping gene, *gyrB*. 

### 5.5. Quantitative Real Time PCR

*Staphylococcus aureus* strains from children diagnosed with atopic dermatitis were cultured in TSB to mid-exponential growth and then stimulated with recombinant type I AIP (50 nM) with and without the test material. After 3 hours, cells were lysed by the addition of Lysostaphin (Sigma-Aldrich, St. Louis, MO, USA) together with lysozyme, and then mRNA was collected using the E.Z.N.A. Bacterial RNA Kit (Omega Bio-Tek, Norcross, GA, USA). Quantitative real time PCR (qRT-PCR) was performed as described previously [51]. The primers and probes sequences used are shown in Table 2. All probes were labeled with 5′-carbosyfluorescein. Data (*n* = 4 replicates per treatment) were normalized against the *S. aureus* housekeeping *gyrB* gene. 

### 5.6. Statistics

Statistical evaluation was performed using SigmaStat (Systat Software Inc, San Jose, CA, USA) with Mann-Whitney two-tailed t-test for comparison of two groups and one-way ANOVA for the comparison of more than two groups. Multiple comparisons with one-way ANOVA were performed using ad hoc analysis.

## Figures and Tables

**Table 1:** Carnosic acid, carnosol and rosmarinic acid concentrations (*w*/*w*) in rosemary extracts tested in this study. (NA: not available).

Code	Carnosic Acid (%)	Carnosol (%)	Rosmarinic Acid (%)
C1	6.2	NA	NA
C2	11.5	2.8	NA
C3	NA	NA	NA
C4	20.9	2.0	NA
C5	21.29	24.19	1.18
C6	15.29	4.74	0.63
C7	16.39	3.70	6.53
C8	13.42	6.77	6.48
C9	13.83	2.50	6.48

**Table 2:** Oligonucleotides used for qRT-PCR in this study.

Primer/Probe	Sequence
RNAIII forward primer	AATTAGCAAGTGAGTAACATTTGCTAGT
RNAIII reverse primer	GATGTTGTTTACGATAGCTTACATGC
RNAIII probe	FAM-AGTTAGTTTCCTTGGACTCAGTGCTATGTATTTTTCTT-BHQ
psmα forward primer	TAAGCTTAATCGAACAATTC
psmα reverse primer	CCCCTTCAAATAAGATGTTCATATC
psmα probe	FAM-AAAGAVVTCCTTTGTTTGTTATGAAATCTTATTTACCAG-BHQ
gyrB forward primer	CAAATGATCACAGCATTTGGTACAG
gyrB reverse primer	CGGCATCAGTCATAATGACGAT
gyrB probe	FAM-AATCGGTGGCGACTTTGATCTAGCGAAAG-BHQ
